# The great escape: **Pseudomonas** breaks out of the lung

**DOI:** 10.15698/mic2015.10.234

**Published:** 2015-09-23

**Authors:** Angelica Zhang, Stephanie M. Rangel, Alan R. Hauser

**Affiliations:** 1 Department of Microbiology/Immunology, Northwestern University, Feinberg School of Medicine, Chicago Illinois, USA.; 2 Department of Medicine, Northwestern University, Feinberg School of Medicine, Chicago Illinois, USA.

**Keywords:** Pseudomonas aeruginosa, ExoS, pneumonia, dissemination

## Abstract

The Gram-negative bacterium Pseudomonas aeruginosa is a major cause of hospital-acquired infections and the focus of much attention due to its resistance to many conventional antibiotics. It harbors a wide range of disease-promoting virulence factors, including a type III secretion system. Here we review our recent study of ExoS, one of the effector proteins exported by this type III secretion system. Using a mouse model of pneumonia, we showed that the ADP-ribosyltransferase (ADPRT) activity of ExoS caused formation of “fields of cell injection” (FOCI) in the lungs. These FOCI represented ExoS-injected clusters of type I pneumocytes that became compromised, leading to disruption of the pulmonary-vascular barrier and subsequent bacterial dissemination from the lungs to the bloodstream. We discuss the potential mechanisms by which these processes occur as well as the novel techniques used to study ExoS function in vivo.

Acute lower respiratory tract infections are among the leading causes of disease burden worldwide and occur frequently in both community and healthcare settings. Several bacterial species are particularly adept at overwhelming the defenses of the airways and lungs. The net result is persistence of bacteria in the pulmonary environment and an ongoing but only partially effective inflammatory response that causes collateral damage to lung tissues. Interestingly, bacteria sometimes breach the pulmonary-vascular barrier and disseminate from the lungs to the bloodstream. Clinically, this development warrants close monitoring and is associated with increased mortality, but the mechanisms by which it occurs are still being elucidated.

To investigate how bacteria disseminate from the lungs to the bloodstream during pneumonia, we chose to study the Gram-negative bacterium *Pseudomonas aeruginosa*. This pathogen, which is a leading cause of ventilator-associated pneumonia, uses a type III secretion system (T3SS) to inject effector proteins such as ExoS directly into the cytoplasm of host cells. With regard to pneumonia, three aspects of ExoS are particularly important. First, the gene encoding ExoS is present in approximately 70-80% of *P. aeruginosa* clinical isolates. Second, ExoS enhances the severity of disease and the frequency of dissemination to the blood in mouse models of pneumonia. Third, substantial effort has led to a detailed understanding of the molecular activities of ExoS. These features made it an ideal candidate for studying how *P. aeruginosa *escapes from the lungs.

As mentioned, much is known about the molecular mechanisms by which ExoS intoxicates host cells. ExoS is a bifunctional toxin containing both ADP-ribosyltransferase (ADPRT) and GTPase activating protein (GAP) activities. Through the T3SS apparatus, ExoS is translocated into mammalian cells where it adversely affects a variety of cellular processes. In cell culture experiments, both ADPRT and GAP activities of ExoS disrupt the host cell actin cytoskeleton, which in turn contributes to loss of cell-cell adhesion and inhibition of phagocytosis.

Much less is known about how ExoS is wielded by *P. aeruginosa in vivo* to cause severe pneumonia. Our group had earlier shown that ExoS ADPRT activity inhibited phagocytosis of bacteria by neutrophils in a mouse model of pneumonia. Since neutrophils comprise an important part of the early defense against *P. aeruginosa*, this inhibition contributes to the persistence of bacteria in the lung spaces. In the current study, we expanded these investigations to directly examine the cell types injected by ExoS during pneumonia. As expected, neutrophils were the predominant cell type targeted for injection during early pneumonia, consistent with the ability of ExoS to inhibit phagocytosis by neutrophils. Somewhat surprising, though, was that type I pneumocytes became injected with ExoS later during the course of infection. These injected pneumocytes were observed in localized discrete clusters, which were designated “fields of cell injection” (FOCI). As the infection progressed, the FOCI expanded and contained increasing numbers of dead type I pneumocytes. Since type I pneumocytes comprise the pulmonary-vascular barrier, we hypothesized that ExoS may allow *P. aeruginosa* to breach this barrier and facilitate bacterial escape from the lungs to the bloodstream. Indeed, we found that pulmonary-vascular leakage and bacterial dissemination to the blood and liver of infected mice increased with the size of the FOCI. These findings suggest a new model by which ExoS facilitates *P. aeruginosa* dissemination during acute pneumonia based on FOCI formation (Figure 1).

**Figure 1:**
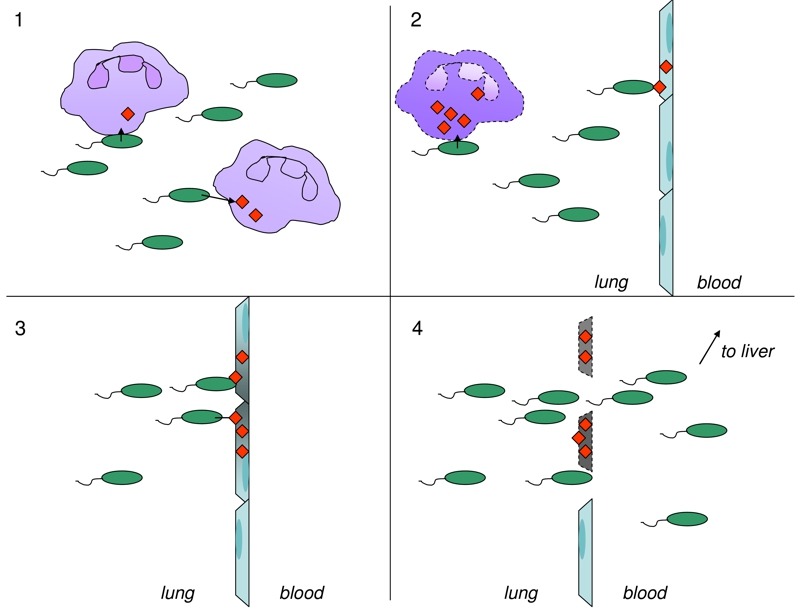
FIGURE 1: Model for ExoS-mediated bacterial dissemination during acute pneumonia. **1.** Early during infection, neutrophils (purple) are injected with ExoS (red), preventing phagocytosis and clearance of *P. aeruginosa *bacteria (green). **2.** Later during infection, bacteria encounter the pulmonary-vascular barrier and inject type I pneumocytes (blue) with ExoS. **3.** ExoS injection occurs at discrete regions termed FOCI (gray), which expand as infection progresses and type I pneumocytes are killed. **4. **Pulmonary-vascular leakage allows bacteria to disseminate into the bloodstream and to the liver.

Interestingly, the critical steps in *P. aeruginosa *dissemination (expansion of FOCI, type I pneumocytes cell death, and breach of the pulmonary-vascular barrier) were each dependent upon the ADPRT activity but not the GAP activity of ExoS. These findings again demonstrate the importance of ADPRT activity to the virulence of ExoS and suggest a molecular mechanism for FOCI formation.

Among the substrates of the ADPRT domain of ExoS are ezrin/radixin/moesin (ERM) proteins, Ras, Rap1, and Rap2. Modification of each of these could conceivably contribute to FOCI formation. When phosphorylated, ERM proteins are activated and serve as linkers between the actin cytoskeleton and plasma membrane-anchored proteins. In addition they also regulate Rho GTPases to control adhesion, actin cytoskeletal arrangement, and apoptosis. Together, these interactions allow ERM proteins to stabilize the actin-rich cell cortex and adherens junctions, which in turn maintains the epithelial barrier. The addition of an ADP-ribose moiety to ERM proteins by ExoS prevents their phosphorylation, which results in loss of membrane integrity.

Another major substrate of the ExoS ADPRT domain is the small GTPase Ras. Ras signals through major pathways involved in cell growth, proliferation, survival, and differentiation. ExoS ADP-ribosylation of Ras prevents efficient binding to the Ras GNEF Cdc25, causing a slower rate of nucleotide exchange. This in turn uncouples Ras signal transduction and results in apoptosis of the host cell. Finally, the Ras-related GTPases Rap1 and Rap2 are involved in cellular adhesion to the extracellular matrix and in maintenance of cell-cell junctions. ADP-ribosylation by ExoS blocks these functions. Thus the ADPRT activity of ExoS may cause FOCI formation and subsequent bacterial dissemination by targeting and disrupting multiple host cell signaling pathways.

Discovery of ExoS injection into type I pneumocytes and the formation of FOCI would not have been possible without two novel techniques. First is the adaptation of the β-lactamase/CCF2-AM reporter assay system for use within an entire intact lung rather than isolated cells *in vitro*. This approach allowed the visualization of lung sections and the identification of not only ExoS injection of phagocytic cells but also of type I pneumocytes. The latter cell type is difficult to remove from lungs by mincing or bronchoalveolar lavage and therefore had not been examined in previous studies of type III secretion. Second, we used the recently developed TissueFAXS imaging system (TissueGnostics USA Ltd.) to view entire lung sections from infected mice. This system allowed detection and quantification of different types of ExoS-injected cells within tissue sections of lung lobes. Furthermore, it provided the spatial distribution of injected cells relative to each other and to bacteria, which was instrumental in identifying FOCI. The TissueFAXS technology has tremendous potential in the study of host-microbe interactions within intact tissues. Its ability to combine the spatial aspects of fluorescence microscopy with the quantification of flow cytometry allows complex analyses of *in vivo* specimens. These imaging approaches should be applicable to other pathogenic bacteria and to different organs and tissues.

Our study suggests several future lines of investigation. The possible molecular mechanisms of FOCI formation described above are amenable to testing in cell culture models of infection. It is unclear whether the formation of FOCI is limited to the lungs or whether a similar phenomenon occurs with epithelial barriers in other organs. Also, the toxins of many other bacteria contain ADPRT domains, and it would be interesting to examine whether these toxins also contribute to bacterial dissemination or disruption of epithelial barriers. Finally, identification of the factors that mediate bacterium-type I pneumocyte interaction and lead to ExoS injection into these cells to form FOCI is an important goal and would facilitate the development of inhibitors that blocked these interactions. Such inhibitors could be utilized therapeutically to prevent FOCI formation. In so doing, they would effectively block the escape route used by *P. aeruginosa *to break out of the lungs.

